# Clinical Reflections

**Published:** 1881-03

**Authors:** Ad. Lippe

**Affiliations:** Phila.


### CLINICAL REFLECTIONS.

BY AD. LIPPE, M.D., PHILA.

Mr. C. aged forty-five years, enjoying always good health, living very regular and engaged in large business, complained on the 12th of November, 1880, he felt sick all over, better when at rest, stiffness of the limbs, nausea with headache and poor appetite; received one dose of *Bryonia* C.M. (Fincke). Feeling better, he followed his usual occupation but was compelled to come home in the afternoon of the 18th of November and take to his bed with a chill which was followed by high fever, cold feet, very hot head, flushed face, and, contrary to his habit, wishing to lie with his head high. Pulsating headache, pulse 96 per minute urinary secretion almost suspended, some nausea, very little thirst but great weakness. One dose of *Belladonna* C. M. (Fincke) was adminstered 7 P.M. Had a much disturbed night, many dreams and visions, slight delirium, more thirst, with scanty secretion of very dark urine, headache continues, tongue clean. On the evening of the 19th of Nov. his pulse was 106 per minute; he complained of feeling bruised all over, motion greatly increased this soreness; coughed at times, and then complained of stitches in both sides of the throat, did not feel inclined to sleep, headache was less severe, thirst increased, he wanted large quantities of water at a time, otherwise no change. At 7 P.M. he took one dose of *Bryonia* C.M. (Fincke). Nov. 20th. Had a very restless night, changing his position frequently. Slight delirium. The pain in the throat better; so was the bruised feeling; urinary secretion unchanged; skin very dry; thirst less; this condition continued all day. When asked why he changed his position so frequently, he said that he did so in order to relieve pains which increased during continuation in one position; that he felt better after such a change of the painful position till he had occupied it for some time. Pulse 120 per minute. He recieved one dose of *Rhus tox* C.M. (Fincke) at 6 P.M. to be repeated if he did not perspire by 9 P.M. Nov. 21st. At 7 P.M. of the 20th his skin became moist; by 8 P.M he was in a profuse perspiration, and from that time he began to feel better. The urinary secretions gradually increased, leaving an increasing deposit of phosphates. The perspiration continued till the 23d. He showed no desire for food or drink but cold water or an occasional glass of milk. The pulse was less frequent and soft. As there was an apparent pause in the improvement on the 24th of Nov. (the 7th day of the disease) he received another dose of *Rhus tox.* 50 M (Fincke) at 8 P.M. Nov. 25th. Perspired very freely all night and asked for some light food. Soft-boiled eggs and toast. This food tasted good; all his symptoms gradually improved day by day without further medication. Urine became profuse and clear. On the 30th he sat up and enjoyed a full dinner, asked for his favorite Burgundy wine and felt well. During these two weeks he had no movement of the bowels and his first evacuation—perfectly natural—came on the 1st December. On the 3d of December he rode out in a carriage and returned for a short time to his country house. On the 10th of December, though otherwise very well, he complained of a slight return of itching hærmorrhoids which he had had years ago. One dose of *Sulphur* 21 M (Skinner) relieved him at once; since then he has been perfectly well.

COMMENTS: To all appearances this was a grave case of disease, and might be called a case of typhoid fever. The patient fully recovered under strictly homœopathic treatment without any resort to auxiliary and supplementary means, such as of late have been recommended in grave cases. The law of the similars and Hahnemann's advice how to apply that law were our only guide. The most difficult part of the treatment of this case was the finding of the cause of the distressing and increasing restlessness; had we not patiently and diligently examined the sick, had we hastily given him arsenicum for this restlessness the much desired early crisis by perspiration would not have come to the rescue. After this important symptom—worse when lying for a time in the same position and relief when that position was changed, had been ascertained it was easy enough to see the remedy. Patients do not often give us the symptoms as we would wish them given, and we have then to apply our individual judgment to find by interrogation what the real, true, symptoms of the sick are. But we must never rest till we obtain a clear conception of the case before us.

Hahnemann tells us in pargraph 4 of "The Organon of the Healing Art" that he, (the true healer) is also a health preserver if he learns to know what causes health disturbances, and what creates and supports diseases and when he learns how to remove these causes.

In the above case the question arose "Why was he sick?" A man who lived a prudent and regular life, who had not been exposed to the fever miasm of any malarial district could not well sicken without cause. His residence was a well-built house; there were no fixed washstands in it, it was well ventilated and scrupulously clean, even the cellars being very clean. After a pains-taking examination it was found that back of his counting room existed a faulty wall and on rainy days the odor from it compelled the occupants of the counting room and large store to close the windows. As soon as this discovery was made Mr. C. took much pains to ascertain the true condition of things, and at once applied the proper remedies for the removal of this disease-creating nuisance.

Mr. T., twenty-six years old, always well, having had but one attack of pneumonia three years ago (under allopathic treatment) was taken sick on Dec. 31st, 1880, and retired early. He passed a very bad night, and requested advice early on the morning of Jan. 1st, 1881. He had tossed about his bed all night without sleep, slight stitches in his sides, no cough, much thirst, and felt very much distressed, pulse 96. On auscultation and percussion nothing abnormal was observed, and it seemed to be a case of pleurisy. One dose of aconite C.M., was given at 8 A. M. At 6 P. M. he complained of great dispnœa, violent stitches when taking a long inspiration, much aggravation on motion. Has taken nothing but water all day. When moving he coughs very hard and suffers much pain in the lungs. Pulse 120 per minute, face flushed, head hot and painful. Received one dose of *Bryonia* C.M. At 10 P. M. he began to perspire profusely, and continued to do so for thirty-six hours. The cough became loose, his appetite returned, and on the 4th of January he was well enough to leave his room and go to a friend's house. He has been perfectly well ever since.

COMMENTS: Here we had a clear case of Pleuro-pneumonia, a much dreaded disease on account of the great mortality under allopathic treatment. As it often happens, so in this case, silly and ignorant friends looked dispairingly at the simple and plain treatment. There was, in their opinion, nothing done for the sufferer; he surely ought to have a fly-blister clapped over his chest, or should be bled at once, or something energetic should be done to rescue him from certain distruction. Because absolutely nothing was done for him, even an auxiliary mustard plaster being rejected, and a supplementary mustard footbath not being tolerated at all, despairing, but ill-informed friends left him for the night with regrets that so fine a fellow as he was should so stubbornly reject the means he had seen used before to no good purposes but only to be followed by evil and sad results. When, on the second morning, these anxious friends came and were told how much better he felt; when, on the third morning they found him gobbling up a large and luxurious breakfast they were compelled either to own up to the great success of homœopathy, or do, as they often do, take the liberty of declaring that they were mistaken, that he really was not much sick after all, else he could not have so speedily recovered. Nevertheless, they would have blistered him, and now blistered are their tongues for violating common sense, and perverting ordinary logic in order that they may in future keep on plodding along in darkness, and see mankind tortured by unscientific boluses, blisters and physics; and what will pretenders learn from such a case, pretenders who fly to *aconite* and *Belladonna* in alternation because, forsooth, there is fever! Just as much as the fly-blister-worshipers learned in this case—nothing. There are none so blind as those who do not wish to see.

